# Dentin tubule obturation of a bioglass-based dentin desensitizer under repeated exposure to lactid acid and brushing

**DOI:** 10.1186/s12903-019-0962-7

**Published:** 2019-12-05

**Authors:** Andrea S. Manz, Thomas Attin, Beatrice Sener, Philipp Sahrmann

**Affiliations:** Clinic of Conservative and Preventive Dentistry Periodontology and Cariology Center of Dental Medicine, University of Zuric, Plattenstr, 11 8032 Zurich, Switzerland

**Keywords:** Dentin sensitivity, Hypersensitivity, Bioglass, Desensitizer, Electron microscopy

## Abstract

**Background:**

Dentin hypersensitivity is a frequent finding especially in periodontitis patients. Conventional treatment aims for obstruction of dentin tubules by disabling liquid and osmotic fluctuation to and from the pulpal chamber. A novel bioglass-based desensitizer was shown to obstruct tubules and to resist periodic exposure to lactic acid. Whether this obstruction is resistant to brushing had not been tested so far. Accordingly, the present study aimed to assess dentin tubule obstruction after repeated acid exposure and brushing.

**Methods:**

Sixty dentin discs were cleaned with 17% EDTA, mounted into a pulp fluid simulator and randomly divided into 3 groups: No surface treatment in Group A, Seal&Protect® in group B and DentinoCer in group C. Discs were exposed to 0.1 M non-saturated lactic acid thrice and standardized brushing twice a day for 12 days. At baseline and after 2, 4 and 12 d samples were removed from the setting and prepared for top-view SEM analysis to assess tubule obstruction using the Olley score. Discs were then vertically cut and the section surface morphologically assessed using backscatter imaging. For both vertical and sectional surfaces EDX analysis was used to characterize the surface composition in the tubular and inter-tubular area.

**Results:**

Group A showed clean tubular lumina at all time points. From day 2 onwards dentin showed exposed collagen fibers. Group 2 initially showed a complete surface coverage that flattened out during treatment without ever exposing tubules. At baseline, samples of Group C displayed a complete homogeneous coverage. From day 2 on tubules entrances with obstructed lumen became visible. While on day 4 and 12 the dentin surface exposed collagen fibers the lumina remained closed. EDX analysis of the vertical and horizontal views showed that P and Ca were predominant elements in both the inter- and tubular dentin while Si peaks were found in the tubule plugs.

**Conclusion:**

While group B displayed a packed layer on the surface during the whole investigation time group C samples lost their superficial layer within 48 h. Tubule plugs containing considerable Si proportions indicated previous presence of DentinoCer, while high Ca and P proportions suggest obturation by dentin-like material.

## Background

Dentin hypersensitivity (DH) is a very common problem in daily practice: Epidemiologic studies report a rather broad range of a general prevalence between 3 to 98% [1, 2]. The large variance is a consequence of different cohorts in the single studies, with young and healthy patients on one side and patients with an especially high risk due to certain preconditions like multiple tooth erosions, pronounced tooth wear and patients with loss of periodontal attachment, on the other side [3–5].

DH is defined as a sharp pain of short duration that is caused by thermal, tactile, chemical or osmotic stimuli on tubuli in dentin exposed to the oral cavity, and that cannot be attributed to other reasons [6]. Though these pain sensations are quickly transient it is their high intensity that renders this sensation to be strongly incriminating to concerned patients, and it is effectively reported to negatively affect patients’ quality of life [7, 8] [9].

Several predisposing factors have been reported to enhance risk and intensity of DH in the presence of exposed dentin surfaces. Incorrect brushing techniques like horizontal scrubbing with hard or not-rounded bristles and high-abrasive toothpastes [10–12] [13] in combination with frequent intake of erosive drinks or food [14] seem to play the major role in the pathogenesis of DH.

Accordingly, therapy aims on one hand to eliminate risk factors and – on the other - to suppress the trigger for the pain sensations [15]. In absence of a clinically detectable dentin defect, therapy aims for impregnation or sealing of the porous dentin surface. On this behalf so-called desensitizers are used [5, 16]. Products with high wettability enter the orifices of dentin tubuli and penetrate into their lumina up to several hundreds of μm of depth [17, 18]. Though in the first instance definitively effective, modern desensitizers suffer from two important shortcomings:

First, though initially effective, pain relief tends to fade yet after several weeks [19]. This is especially the case, if the previously specified risk factors have not been eliminated and sealed dentin is subjected to continuous abrasion [20, 21].

Second, most modern desensitizers use formulations that contain potentially hazardous components. Molecules like hydroxyethyl methacrylate (HEMA), triethylene glycol dimethacrylate (TEGDMA), camphorquinone and Bisphenol-A-glycidyl-dimethacrylate (BisGMA) which precipitate in the dentin tubules remain within the host and have been shown to interfere with health, namely directly by cytotoxicity [21], or – more complex – due to their allergenic [22, 23] or mutagen [23] effects and estrogen-like activity [24].

In order to get over this problem newer products rely on less hazardous products. Bioglasses mainly contain biocompatible substances like oxides of silicium, calcium, sodium and phosphate [25, 26]. Still, little is known regarding the potential to obturate dentin tubules with bioglasses [27] and the stability of such obturation when exposed to bacterial products and oral hygiene measures.

Recently, a novel bioglass desensitizer based on a soluble calcium phosphate bioglass embedded in a slightly alkaline gel was tested in-vitro. Surface analysis of dentin disks in an experimental set-up simulating pulp fluid pressure and bacterial acid attack showed complete coverage of the surfaces, that previously showed free tubule orifices, over 12 d of intermittent exposure to 0.1 M unsaturated lactic acid [28]. Though the material thereby showed an important prerequisite for its potential clinical use, nothing is known about how resistant a superficial layer of the regarding bioglass matrix layer and a deeper portion of obturated tubular dentin might be, if subjected to the mechanical stress of tooth brushing and if applied together with intermittent exposure to acid. Yet it was shown that a bioglass (45S5 paste) might provide permanent coverage of enamel surfaces and promising decrease of dentine permeability after 6000 cycles of standardized brushing [29, 30], but there are substantial differences regarding the experimental design of the present study and the composition of the assessed bioglass.

Therefore, it was the aim of the present investigation to assess the obturating effect of a novel bioglass based desensitizer on dentin surfaces, when samples were exposed to both, periodic brushing and recurrent acid exposure, in order to better simulate the clinical situation in the oral cavity more exactly.

## Methods

### Preparation of dentin specimens (Fig. 1)

Dentin discs were harvested from fresh bovine incisors. Incisors were taken from cow mandibles that were purchased from the Zurich abattoir, where the animals had been slaughtered in the morning of the same day. The incisors were prepared as described to detail elsewhere [28]. In brief, 60 round dentin discs with a diameter of 3.0 mm were embedded in the centre of a round methacrylate frame of 5.0 mm diameter. Care was taken to preclude any resin contamination of the flat surfaces of the dentin discs. Composed specimens were grinded to a thickness of 1.5 mm and gradually smoothened using 2′000 grit and 4′000 grit abrasives (Struers waterproof SiC, Birmensdorf, Switzerland) on a water-flushed grinding wheel (Struers, Tegramin-30). Afterwards, specimens were cleaned in an ultrasonic bath of 17%-EDTA for 10 min and then gently brushed with a soft toothbrush (Curaprox Supersoft, Curaden, Dietikon, Switzerland) under abundant tab water for another minute. The moist dentin samples were exposed to gamma radiation at 12 kGy for 34.6 h in order to minimize the risk of bacterial overgrowth during the investigation period. During the whole preparation process samples were stored in sterile water to avoid exsiccation.

**Fig. 1:**
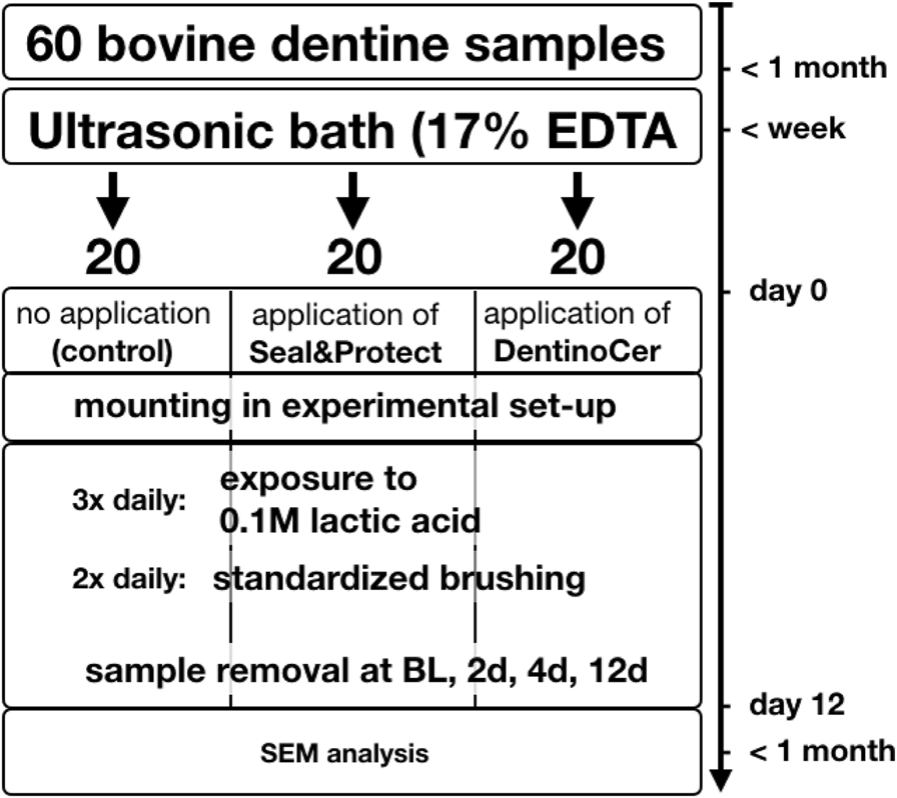
Flow-chart of sample preparation and treatment

#### Set-up of the experiment (Figs. 1 and 2)

**Fig. 2:**
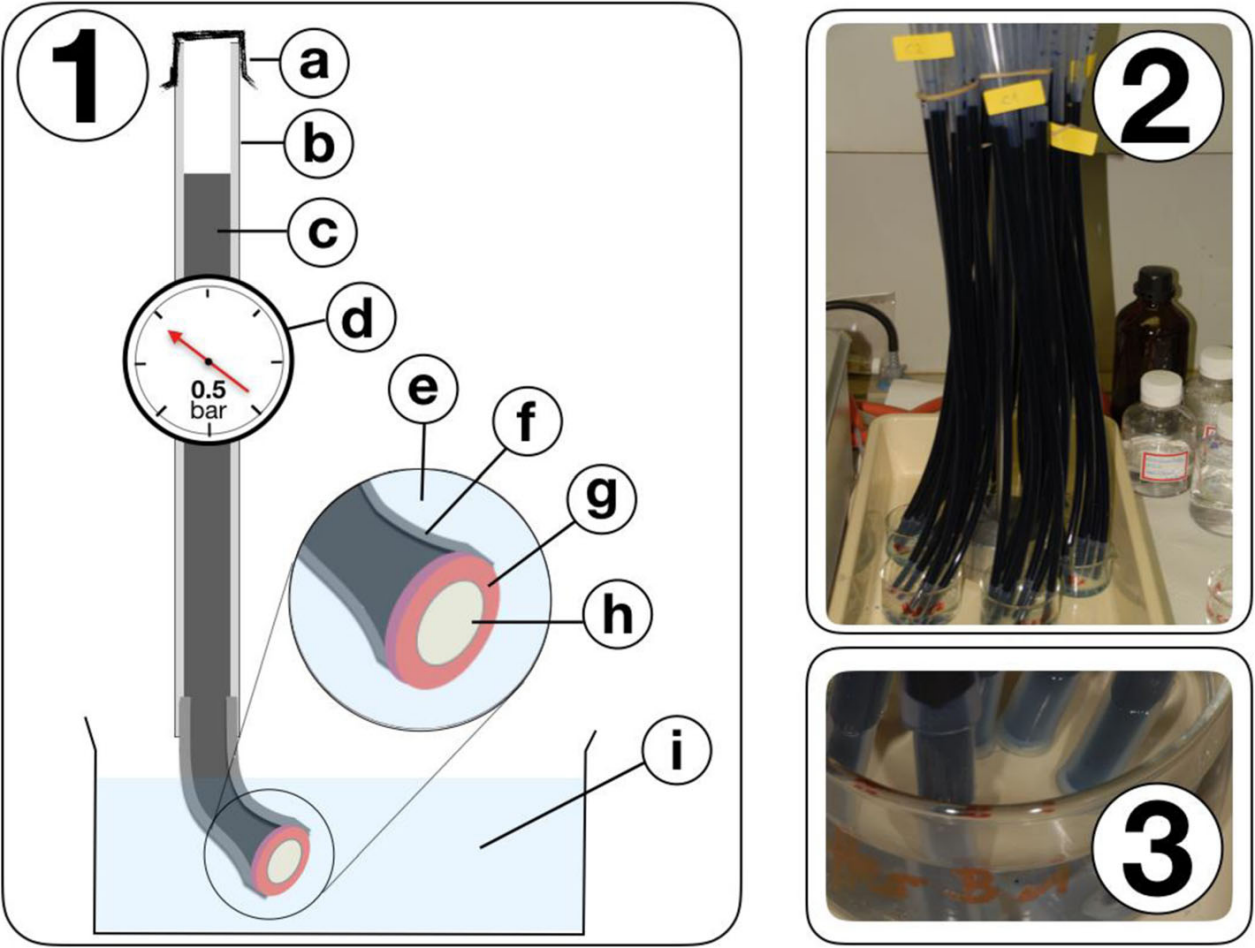
Experimental set-up. 1 a – tube cover, b – pvc-tube, c – artificial saliva, d – adjusted pressure 0.5 bar, e – tap water, f – silicone tube, g – methacrylate socket, h – dentin disc, i – water basin. 2 Photo of the pendulous pvc tubes. 3 Silicon tube tips in the water basin

As previously described and then modified [28, 31], 60 PVC tubes of 0.6 m length and an internal diameter of 7.0 mm were gas-sterilized with ethylene oxide (3 M Schweiz, Rueschlikon, Switzerland) for 24 h. For the simulation of pulp fluid pressure, the tubes were vertically mounted. Into the lower endings of each tube another 4 cm long flexible silicone tube with an internal lumen of 5 mm was mounted, and the lower opening was closed with one dentin specimen each, using a special device that allowed for insertion without touching the flat disc surfaces [28]. Tube endings together with discs were stored in water basins that were placed under the PVC tubes (see Fig. 2). The latter were then filled with artificial dentin fluid (ADF) to a height of 0.5 m, thereby simulating an outbound pulpal fluid pressure of 0.5 bar. All ADF components had previously been ultra-filtrated to further minimize the risk of bacterial overgrowth (Filtropur VSO 0.2, Sarstedt, Nuermbrecht, Germany). For the same reason, the experimental set-up was closed under a laboratory hood during the whole investigation period, Each person manipulating the set-up wore disinfected medical gloves, a surgical mask and – if applicable – had the hair tied up.

#### Pre-treatment of specimens

Sixty dentin discs were randomly assigned into three groups of 20 each.

A. (control group): No treatment of the dentin surface.
B. (Seal&Protect®): The surface of each specimen was air-dried and Seal&Protect® (Densply Sirona, Baden, Switzerland) was applied according to the manufacturer’s manual. For the application, a sterilized single-use applicator (Orbibrush, Orbis, Muenster, Germany) was used. After a residence time of 20 s with a gentle excess film on the surface, samples were air-dried for 5 s and light-cured at a wavelength of 385–515 nm (1200 mW/cm2) from a distance of 5 mm for 10 s (bluephase G2, Ivoclar Vivadent AG, Schaan, Liechtenstein). In a second step, Seal&Protect® was applied and cured again in the same mode.
C. (DentinoCer, Table 1): Sample surfaces were air-dried and DentinoCer (Biocer, Bayreuth, Germany) was applied with a sterilized single-use applicator (Orbibrush, Orbis, Muenster, Germany) for 5 min, and stayed for another 5 min until excess material was removed by airflow.

**Table 1:** Composition and pH-value of the dry weight of DentinoCer®

Component	Dry weight %
Ca	20.4
P	31.2
Si	4.55
pH	6.6–6.7

Both applied test liquids had previously been subjected to gamma-sterilization (23 kGy).

After application, the samples at the tube endings were put back into the water basins.

#### Exposure to lactic acid and brushing

For the exposure to lactic acid for three times per day, tube endings with the dentin samples were taken out of the water basins and placed into buffered sterile lactic acid (pH 5) for 10 min. Then, samples were rinsed with sterile tap water and put back into the basins.

**Fig. 3:**
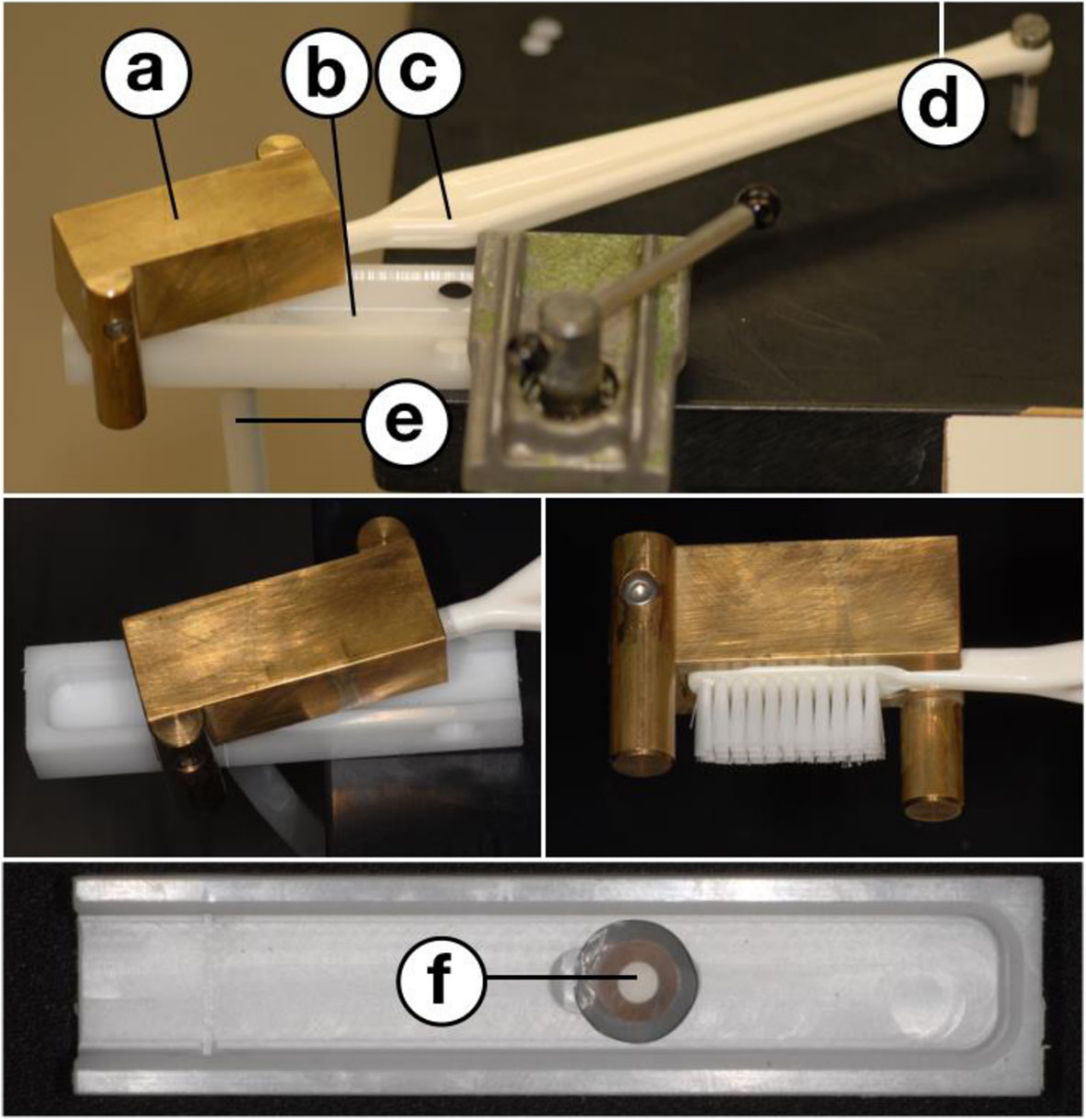
Set-up for standardized brushing after exposure to lactic acid. **a** – weight block (200 g) with lateral guide bar (not reaching the ground). **b** – brushing chamber. **c** – toothbrush (Curaprox Supersoft, Curaden, Dietikon, Switzerland. **d** – fulcrum of the toothbrush. **e** – tube with artificial dentin fluid. **f** –dentin sample in the chamber base

After the first and the third exposure per day to lactic acid samples in the tube endings were placed into a guide rail that allowed for brushing with a standardized force of 200 g (see Fig. 3). Each time, samples were subjected to 10 brushing strokes by a brush with rounded medium nylon bristles (Paro M43, Paro AG, Subingen, Switzerland) [32, 33]. Exposure to lactic acid and brushing was performed each day until day 12.

From each group five baseline samples were removed from the set-up immediately after pre-treatment in the test groups. Another five samples of each group were removed at day 2, 4 and 12. Before further progressing for SEM assessment samples were placed in 2.5% glutaraldehyde.

#### Imaging

Specimens were fixed in 2.5% phosphate-buffered glutaraldehyde solution for 1 d. After rinsing for 3 times in phosphate buffer solution, samples were exsiccated in an ascending ethanol row (50–96%). Samples were then saturated for 72 h and fixed in photo-curing one-component methacrylate-based resin (Technovit 7200 VLC, Haereus Kulzer, Hanau, Germany). Samples were then placed on SEM holders and got sputter-coated with a standardized gold layer of 8.0 nm (Sputter CCU-010, Safematic GmbH, Bad Ragaz, Switzerland).

A surface assessment was performed at 10 kV (Zeiss Supra 50 VP, Zeiss, Oberkochen, Germany). Photos were taken at 1000x and 10,000x magnification. Images were taken from a standardized area (300 μm to “right” and “above” from the discs’ central point) Assessment of dentin tubules obturation was performed using the Olley score with values from one to five indicating the degree of occlusion (1 – occluded, 2 – partially unoccluded, 3 – equally occluded/unoccluded, 4 – partially occluded, 5 – unoccluded) [34].

#### EDX-analysis

In order to characterize the material that obturated the dentin tubules of DentinoCer specimens, energy-dispersive X-ray spectroscopy (EDX, Zeiss Supra V50, Carl Zeiss, Oberkochen, Germany) was used. Proportions of different chemical elements of interest were detected and reported as percentage of the whole composition. The respective analysis was performed from two directions, top view and vertical intersections. For the latter, specimens were cut centrally (Buehler, ISOMET® low speed saw, Prüfmaschinen AG, Dietikon, Diamant Cut-off Wheel, Struers GmbH, Birmensdorf, Switzerland) and polished.

On this behalf, the samples were newly embedded in resin and vapor-coated with coal powder which allowed for a better discrimination by backscatter analysis. Photos of the intersections were made at 10 kV (Zeiss Supra 50 VP) and at a magnification of 10.000x. Element analysis was performed from the tubular plugs and – as a control - from intertubule dentin areas.

Imaging was performed by a single operator (BS) who was unaware of the pre-treatment of the specimen. Likewise, the person analyzing the respective images (PS) was blinded to the group allocation.

## Results

### Surface analysis

#### Group a (control)

**Fig. 4:**
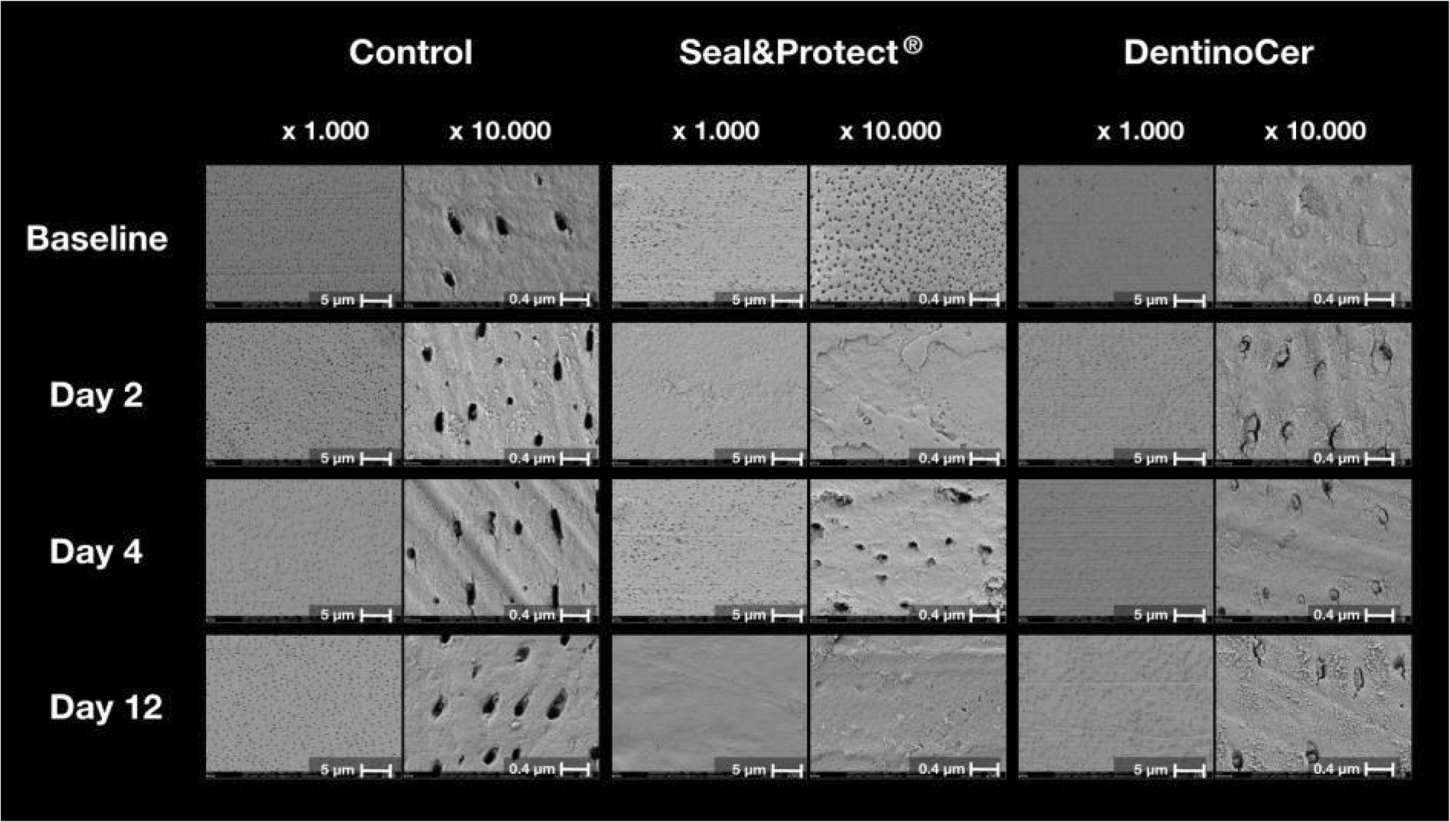
SEM images at 1′000 and 10′000x magnification of typical samples from the 3 groups at different time points

Top view images at baseline generally showed open tubule apertures throughout the samples at 1000x and 10,000x magnification (Fig. 4). After 2, 4 and 12 days, tubular lumina appeared to be clean. After 12 d sporadically some fluffy particles were visible at the orifices’ inner margin, without noteworthy obstruction of the respective lumina (Fig. 4). From day 2 on, non-tubular dentin surfaces displayed parallel brushing furrows with a distance of 500–1000 nm at a magnification of 10′000x and single fluffy particles appeared on the sample surfaces.

At all points of time, the control samples with generally open orifices were rated with an Olley-Score of 5 (Table 2).

**Table 2:** Top view occlusion assessment of dentin tubule

Group	baseline	2d	4d	12d
Controls	5	5	5	5
Seal&Protect	1	1	1	1
DentinCer	1	1–2	1–2	1–2

Olley scores: 1 – occluded, 2 – partially unoccluded, 3 – equally occluded/unoccluded, 4 – partially occluded, 5 – unoccluded [34]. Values in brackets were found very sporadically

#### Group B (Seal&Protect®)

Baseline samples show a crispbread-like porous surface at 10,000x magnification. The surface is characterized by a homogeneous granular texture with round pores of a diameter of ca. 50 nm. From day 2 on, this surface got progressively abraded and pores vanished from day 2 on. At later time points, some samples exposed round or amorphous pores of a much larger diameter (200–300 nm), which in the course of further brushing, disappeared again. On day 12, the surface appeared completely flat and homogeneous. Brushing furrows were visible on several samples at different times (Fig. 4).

With completely occluded dentin tubules, the respective Olley score for each time point was 1 (Table 2).

#### Group C (DentinoCer)

Top view images of the samples treated with DentinoCer displayed a homogeneous, flat surface at baseline with sporadic drying cracks of a length of about 2 μm and a width of about 0.1 μm. Tubule openings are covered at baseline, but their contours became visible from the second day on. The lumina, however, remained largely filled with a compact substance till the end of the complete observation period. The surface between the tubules appeared identical to the respective areas of the untreated discs of group A, therefore displaying dentin surfaces with brushing furrows and cottony surface areas (Fig. 4).

Olley scores for day 12 range between 1 and 2 (Table 2).

#### EDX analysis

**Fig. 5:**
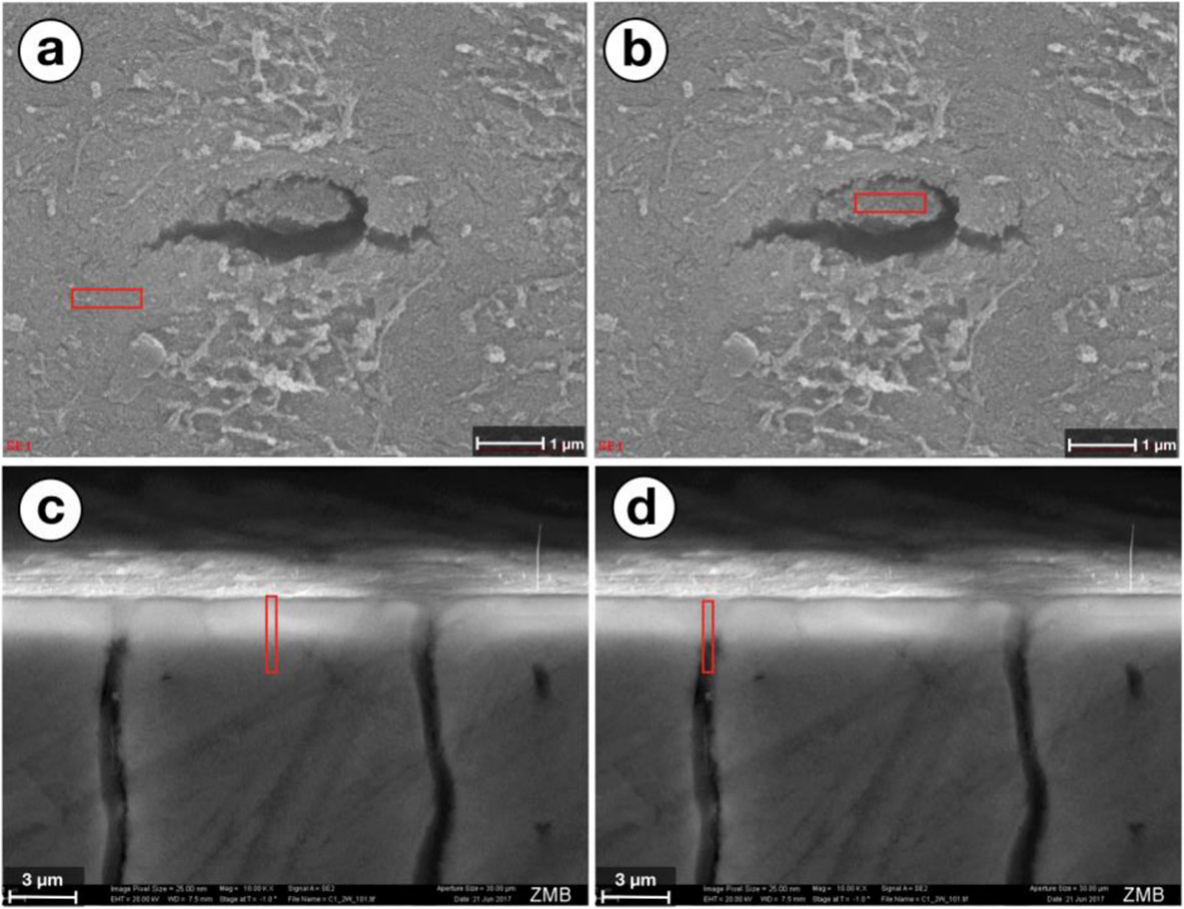
Images from EDX analysis with indicated area of interest. **a** – Top view at 30′000x magnification, area of interest on intertubular dentin. **b** – Top view at 30′000x magnification, area of interest on tubular plug. **c** – Vertical cut view at 10′000x magnification, area of interest on intertubular dentin. **d** – Vertical cut view at 10′000x magnification, area of interest on tubular plug

Element analysis of the DentinoCer samples from top view showed that P and Ca were the predominant elements in both, the plugging material of the tubular orifices and intertubule dentin sections. In the obliterated tubules however, considerable amounts of Si (9–9 ± 2.7%) were found while the intertubule sections were shown to hardly harbor any (0.7 ± 0.2%) (Fig. 5, Table 3).

**Table 3:** Weight percentage of different elements as assessed by SEM from top view

Weight %	Si	P	Ca	O
Intertubular dentin	0.74 ± 0.19	23.48 ± 0.20	50.07 ± 0.75	25.60 ± 0.67
Tubular plug	10.8 ± 2.73	18.18 ± 1.62	39.64 ± 4.90	34.19 ± 4.64

All values given as means ± standard deviations

EDX analysis of the vertical intersections showed, likewise, predominantly Ca and P and considerable amounts of Si (2.3 ± 1.7%) (Fig. 5, Table 4).

**Table 4:** Weight percentage of different elements as assessed by SEM from vertically cut surface

Weight %	Si	P	Ca	O
Intertubular dentin	0.26 ± 0.08	17.28 ± 0.87	36.02 ± 1.94	46.44 ± 2.74
Tubular plug	2.31 ± 1.37	17.67 ± 1.37	35.32 + 3.03	45.04 ± 4.08

All values given as means ± standard deviations

## Discussion

A novel bioglass-based dentin desensitizer has recently been shown to form a stable matrix on dentin surfaces that outlasted repeated exposure to lactic acid over at least 12 d and seemed to obliterate dentin tubules in an in-vitro experiment [28]. Since its performance under mechanical stress as applied by brushing has not been studied so far, it was the aim of the present study to investigate its potential to close superficial dentin tubules in a set-up that simulates pulp fluid flow, periodic exposure to lactic acid and repeated brushing with standardized parameters.

After a twofold application which resulted in a complete and homogenous coverage of the dentin surfaces in terms of an Olley score 1, the matrix layer of DentinoCer got lost during the first 2 days of brushing. The intertubular surface was identical to the respective areas of the untreated surfaces of group A. The exposed cotton-like particles are likely to be parts of collagen that had been exposed due to acid exposure and subsequent brushing. Parallel furrows seem to be due to brushing of the demineralized dentin surface. Though the contours of the tubular orifices became visible, the lumina of the tubules were almost completely obliterated by a rather homogeneous and dense material. The orifices remained obliterated over a period of 12 days, including - on the whole - 36 cycles of exposure to lactic acid for 10 min each and 24 cycles of standardized brushing of ten brushing strokes each with 200 g of loading force. This finding is in accordance with a study of Bakry et al. who tested a different bioglass based on phosphoric acid on dentine and subjected the new surface to a whole of 6000 brushing cycles. The latter study, however, did neither simulate pulpal pressure nor lactic acid exposure [29]. What is more, another study by the same group showed, that the occluding effect of bioglass 45S5 may be improved if the surface is treated with a pulsed CO2 laser at a wavelength of 10.6 μm at 100 Hz [35]. Likewise, another study using 45S5 bioglass on enamel previously showed considerable resistance to (1% citric) acid exposure over an examination period of 18 min [36].

Material that obliterated the tubules turned out to be of a different chemical composition as compared to dentin from intertubular areas, consistently displaying considerable amounts of Si in the EDX analysis. With a proportion of around 4.5% of Si in the dry weight, DentinoCer is the only material with a considerable Si share in the experimental setting. Therefore, its presence proves that DentinoCer had reached the tubular openings after application at baseline and was – in part – still present at day 12 of the experiment, when the plugs emerged in the tubules. *De-novo* presence of obturating material rich of Ca and P compounds on one hand and the visual proof of obliteration on the other hand seems rather close to the original idea of bioglass-related regeneration that has been described for bioglass use, originally in bone [37]. For dentin, bioglass-based materials have so far been reported to show regenerative potential if used as experimental resin based adhesive on dentin discs [38] or in a three-dimensional scaffold in-vitro [39]. In principle, obturation material in dentin tubules, as observed in the present experiment, may derive from different origin: Ca and P components might derive from dentin of the tubular walls. DentinoCer, however, with a nearly neutral pH of 6.7, renders dissolution of ions from neighboring hard tissues rather improbable. On the other hand, dentin might become abraded from the outer dentin surface during brushing and end up in tubule openings. This possibility, however, is not very likely due to the following observations: Firstly, the amount of particles in the tubules did not seem to change with more brushing sequences. Secondly, negative controls from untreated discs did not show depositions in an amount that might explain tubule obliteration. Finally, Ca and P components might have derived from the DentinoCer formula itself, since the product is rich of Ca and P (around 20% and slightly exceeding 30% in the dry weight, respectively, according to the developer’s declarations). Accordingly, Ca and P ions seem to have precipitated in the tubule openings after previous release from the glass particles in an aqueous, alkaline environment that could develop under the matrix layer [40, 41].

Si was found to a smaller extent in the analysis of the vertical cut than when assessed during the top view onto the dentin surface. Figure 4 a-d shows the measuring field for the EDX analysis from both directions. In the analysis of the vertical slice however the measuring field extended beyond the plug in the tubule opening (Fig. 4d), thereby assessing non-obturated dentin areas in the depth of the tubules. As a consequence, areas that are filled with resin-based fixation material have also been investigated. The latter supposedly contains high amounts of C, and – as a consequence of the proportional assessment - lower Si proportions.

While dentin discs from both treatment groups displayed a homogeneous layer that coated the dentin surface, only the resin-based layer of group B endured the chemo-mechanical stress over 12 days, what was already shown by Wegehaupt et al. in a similar in-vitro study on bovine dentin discs [42]. Newly appearing pores had a very different appearance than the tubule openings and the area between those pores showed another morphology than the “naked” dentin surface of samples from group A. Discs of group C lost their layer which had been defined as bioglass matrix layer in a previous publication [28], quickly. While the exposed dentin surface of the samples treated with bioglass displayed the contours of the dentin tubules, the lumina remained obstructed during the whole observation time. Accordingly, the time span that the matrix layer remained was sufficient to allow for dissolution and mineralization of bioglass components what constitutes the essential reaction mechanism of bioglasses [25]. Vertical slice analysis revealed that the obliterating plugs had a depth of around 2 μm. In an in-vitro study by Bizhang et al. simulated the mean abrasion of dentin due to brushing with similar settings, reporting mean (standard deviation) surface loss of 2.50 (±0.43) μm to 21.03 (±1.26) μm for the sonic toothbrush. Accordingly, such plug might be lost by abrasion after a period of 8 month to 6.8 y, strongly depending on the type of brush, toothpaste and brushing force [8], therefore outlasting the time span of common recall intervals.

There are two potential limitations of the experimental model described for this experiment: First, bovine dentin was used instead of human samples. While Wegehaupt et al. [43]showed that dentin from human molars and from bovine incisors do not to show significant differences in terms of abrasion and erosion, Titley et al. [44]showed the same for bonding shear strength. However, there are certain differences in the micro-anatomy of both structures: Even if the diameter of the lumina was shown not to be significantly different, it seems that there are more pores per square millimeter in bovine incisors than in human molars [45].

Secondly, in the present study samples have been pre-treated in an ultrasonic bath with EDTA, which was important to remove contamination due to the grinding process. In combination with the applied simulated pulp pressure, the set-up was intended to mimic a worst-case scenario, with wide open lumina and pulp liquid fluid flow in the opposing direction, the penetration of the tested desensitizer might be more difficult than in any clinical situation. This might also explain the fact that the penetration depth of the tested bioglass product was considerably lower than in another in-vitro study on a similar bioglass-based desensitizer: Moonesi et al. [39] reported penetration depth of 3.5–25 μm in different settings and application times. In a previous study on DentinoCer, a considerably deeper obturation of the tubules (20–100 μm) was reported [28]. Since that study did not involve brushing, the matrix layer remained for a much longer time of 12 d), providing a longer period of time for the bioglass related process of dilution and mineralization. Brushing abstention after application might therefore have a beneficial effect on the depth of tubular occlusion. Exposure to lactic acid in the present study was adapted to previously published experiments [32, 33] while brushing force and the number of cycles were within a range of different values published in in-vitro studies [46, 47].

Before the background of the promising results future studies should assess the clinical performance of the bioglass-based desensitizer, and define – if possible - application protocols which result in relevant patient benefit regarding dentin hypersensitivity.

## Conclusions

Keeping the considerable limitations of the present in-vitro experiment in mind, a double application of the bioglass-based dentin desensitizer might be likely to provide a closure of the tubules with calcium phosphate particles for a period of at least half a year, therefore potentially outlasting the time span between two recall appointments during common periodontal maintenance therapy. The material therefore seems promising for clinical use, and future clinical studies are needed to verify the degree and durability of a potential pain relief by DentinoCer.

## Data Availability

Datasets for this study are available from the study PI on reasonable request.

## Abbreviations

ADF: Artificial dentin fluid
BisGMA: Bisphenol-A-glycidyl-dimethacrylate
DH: Dentin hypersensitivity
EDTA: Ethylendiamin tetraacetat
EDX: Electron dispersive x-ray
HEMA: Hydroxyethyl methacrylate
SEM: Scanning electron microscope
TEGDMA: Triethylene glycol dimethacrylate
